# Authors' reply

**DOI:** 10.4103/0301-4738.53072

**Published:** 2009

**Authors:** Sanita Korah, Thomas Kuriakose

**Affiliations:** Department of Ophthalmology, Christian Medical College, Vellore, India

Dear Editor,

We thank Ramchandani *et al.*[1] for their valuable comments. At the outset we would like to point out that the purpose of our communication[2] was to report the optical coherence tomography (OCT) findings in chloroquine (CQ) maculopathy, and not the treatment regime followed for this patient. The treatment regime is the prerogative of the treating internist. However, we will try to answer the queries raised.

1. Though hydroxychloroquine (HCQ) is safer than CQ for patients with rheumatoid arthritis, it is significantly more expensive and this was the reason this patient was started on CQ.
2. In our institution, we have a system whereby patients on CQ/HCQ are referred by the rheumatologist for regular 6 monthly ocular examinations. At each review, best-corrected visual acuity, color vision, Amsler grid testing, humphreys field analysis (HFA) 10-2, detailed slit-lamp evaluation and a dilated fundus evaluation is done. We do not, as a routine, perform fundus photography for all these patients.
  This patient developed mild vortex keratopathy in Oct 2004. Her vision, color vision, dilated fundus evaluation, Amsler grid testing and HFA 10-2 field evaluation at that time were normal and remained so till April 2006, when she developed the retinal pigment abnormalities around her macula with early, repeatable paracentral field defects.
3. We agree with you that substituting CQ with methotrexate rather than HCQ was probably the better option. However we, in consultation with the internists, felt that a 3 month trial of HCQ was warranted considering the low toxicity of HCQ as compared to the higher systemic toxicity of methotrexate, the higher cost of the drug as well as the increased number of blood investigations required while on this drug. However, as soon as the changes were found to be progressing (which may have occurred even if we had stopped CQ and converted to methotrexate immediately as CQ toxicity is known to continue even after stopping it),[3] the HCQ was discontinued and methotrexate started.
4. We agree that any screening test would be pointless if intervention is ineffective. However, if the test can detect the condition *before* symptoms and signs appear i.e. at the pre-clinical stage, then it may be a very useful tool.
  The patient we presented, was one with all clinical signs of CQ maculopathy. However, as discussed in the communication, there is evidence to suggest that OCT may be a very useful tool for early detection of maculopathy, even before clinical signs emerge. Discontinuing CQ/HCQ at the earliest sign of maculopathy may lead to clinical improvement.[4] Evidence suggests that, before damage occurs to the photoreceptors and retinal pigment epithelium leading to visual signs and symptoms, accumulation of the drug occurs in the ganglion cells.[5] This would lead to damage and thinning of this layer first, followed by resultant thinning of the nerve fibre layer most easily seen with the OCT at the perifoveal and peripapillary region.
  It would be interesting to find out when the earliest detectable changes occur with OCT in these patients. This would require a prospective study of a large cohort of patients. Documenting that changes occur at all in these patients in the late stages is the first step towards the initiation of such studies.
5. While multifocal ERG (mfERG) may be a very sensitive tool to pick up early changes of HCQ toxicity there is no study comparing its efficacy compared to OCT. In addition, the OCT is more widely available, easier to standardize and much easier to perform compared to the mfERG.
